# An effective method for establishing a regeneration and genetic transformation system for *Actinidia arguta*

**DOI:** 10.3389/fpls.2023.1204267

**Published:** 2023-07-31

**Authors:** Wantian Yao, Lingling Kong, Diya Lei, Bing Zhao, Honglan Tang, Xuan Zhou, Yuanxiu Lin, Yunting Zhang, Yan Wang, Wen He, Mengyao Li, Qing Chen, Ya Luo, Xiaorong Wang, Haoru Tang, Yong Zhang

**Affiliations:** College of Horticulture, Sichuan Agricultural University, Chengdu, China

**Keywords:** *Actinidia arguta*, regeneration system, genetic transformation, tissue cultures, plant growth regulators

## Abstract

The all-red *A. arguta* (*Actinidia arguta*) is an anthocyanin-rich and excellent hardy fruit. Many studies have focused on the green-fleshed *A. arguta*, and fewer studies have been conducted on the all-red *A. arguta*. Here we reported a regeneration and Agrobacterium-mediated transformation protocol by using leaves of all-red *A. arguta* as explants. Aseptic seedling leaves of *A. arguta* were used as callus-inducing materials. MS medium supplemented with 0.3 mg·L^-1^ 2,4-D and 1.0 mg·L^-1^ BA was the optimal medium for callus induction of leaves, and medium supplemented with 3 mg·L^-1^ tZ and 0.5 mg·L^-1^ IAA was optimal for adventitious shoot regeneration. The best proliferation medium for adventitious buds was MS + 1.0 mg·L^-1^ BA + 0.3 mg·L^-1^ NAA. The best rooting medium was 1/2MS + 0.7 mg·L^-1^ IBA with a 100% rooting rate. For the red flesh hardy kiwi variety ‘Purpurna Saduwa’ (*A. arguta* var. *purpurea*), leaves are receptors for Agrobacterium (EHA105)-mediated transformation. The orthogonal experiment was used for the optimization of each genetic transformation parameter and the genetic transformation of the leaves was 21% under optimal conditions. Our study provides technical parameters for applying genetic resources and molecular breeding of kiwifruit with red flesh.

## Introduction

Hardy kiwi (*Actinidia arguta*) is native to China and is abundant in locations such as the northeast and north of the country. It has strong resistance to adverse conditions and is an excellent rootstock material. As a third-generation emergent dual-purpose fruit, it not only has a distinct fruit flavor and is easy to consume, but it also has a large market prospective and development value (Macedo et al., 2023). *A. arguta* is one of the top superfood fruits since it includes more than 20 vital elements and vitamins (Latocha, 2017). It also has several horticultural benefits over conventional kiwifruit, the most notable of which is its excellent cold tolerance (up to -30 °C during dormancy) (Debersaques et al., 2015). The ‘Purpurna Saduwa’ (*A. arguta* var.*purpurea*) is a Ukrainian-bred variety, with red flesh and skin (Lu et al., 2022). Its fruit has the highest anthocyanin content of most kiwis, and anthocyanins are present in all parts of the fruit: skin, pericarp and core (Montefiori et al., 2009; Qi et al., 2011). The kiwifruit fruit flavor is generally better in the red variety, followed by yellow and green (Qi et al., 2020). In recent years, customers have been more interested in red-fleshed kiwifruit (due to its high anthocyanin content) for its antioxidant and health-promoting characteristics (Li et al., 2020). Therefore, the all-red *A. arguta* like ‘Purpurna Saduwa’ is very rare, and it deserves the protection and development of germplasm resources.

The all-red *A. arguta* has grown rapidly in ecological agriculture in recent years with the development of tourist gardens and picking gardens and has wide market potential, thus there is a need for a significant number of seedlings for production. The majority of the production is done through standard seed planting, cuttings, and grafting. Unstable progeny traits, easy segregation of traits, late flowering and fruiting, and long growth cycles are characteristics of seed propagation (Abraham et al., 2010). *A. arguta* cuttings, particularly younger shoots with large leaves and long petioles, are clipped in cutting propagation with a third of the leaves removed, making the leaf cuttings susceptible to physiological and invasive diseases that hinder roots (Huang et al., 2010; Long et al., 2010). Cuttings usually do not live as long as plants grown in other ways, and their survival rate is significantly impacted by the seasons as well as strict environmental requirements. With some seasonal limitations, grafting is typically used as a propagation technique for *A. arguta.* However, the grafting site is vulnerable to disease, and whenever one of the parts has a virus during the grafting process, the virus will be present in the grafted fruit tree (He et al., 2012; Liu et al., 2021). One of the main seedling cultivation techniques is plant tissue culture, which has the advantage of saving time and space and of quickly producing large numbers of high-quality seedlings (Qin et al., 2004; Uchendu et al., 2011). According to prior studies, BA (6-Benzyladenine) was principally used as a cytokinin to induce *A. arguta*, while NAA (Naphthaleneacetic acid), IBA (Indole-3-butyric acid), and IAA (Indole-3-acetic acid) were used as auxins (Xie and Lu, 2003; Liu et al., 2013a; Guo et al., 2014).

In this study, we established an efficient fast-breeding system using different combinations and concentrations of plant growth regulators. An effective genetic transformation system for the all-red *A. arguta* was established by optimizing different parameters of transformation conditions. Our results provide technical support for functional exploration and genetic improvement of *A. arguta*. Furthermore, our study has laid the foundation for research on the molecular biology of the *A. arguta* kiwifruit and for obtaining new varieties with a high resistance to storage and disease.

## Materials and methods

### Establishment of an aseptic rapid propagation system

*A. arguta* var. purpurea cv. ‘Purpurna Saduwa’ was provided by the College of Horticulture of Sichuan Agricultural University. The current-year branches were selected and soaked in washing powder water for 30 min, then rinsed in running water for 2 h. The branches were transferred to an ultra-clean bench, disinfected with 75% alcohol for 15 s, and washed once with sterile water, then disinfected with 0.1%(w/v) HgCl_2_ for 6 min and washed three times with sterile water. Next, the branches were cut into 3-cm long stem segments with buds, and the morphological lower ends of the explants were inoculated onto MS + 1.0 mg·L^-1^ BA + 0.2 mg·L^-1^ NAA medium. Five explants were inoculated per bottle and placed in the culture room for 30 d to obtain aseptic plants.

Aseptic leaves (5 mm x 5 mm) were inoculated on MS (Murashige and Skoog, 1962) medium with different concentrations of NAA, BA, and 2,4-D (2,4-Dichlorophenoxyacetic acid) for induction of calli, and data were recorded after 30 d of culture in the dark. Aseptic leaves (5 mm x 5 mm) inoculated on MS medium with different *PGR* (Plant growth regulator) ratios of tZ (Trans-Zeatin), TDZ (Thidiazuron), NAA, and IAA, were cultured in the dark for 30 d and then under a 16 h light/8 h dark photoperiod for 30 d. The culture-induced adventitious shoots, about 3 cm in height, and these were transferred to MS medium without *PGRs* for 15 days, and then transferred to MS medium containing different hormone ratios of BA and NAA for the proliferation of adventitious shoots for 30 days under a 16-hour photoperiod. Rootless plantlets (3 cm tall) were inoculated on 1/2 MS medium with IBA (0, 0.3, 0.5, 0.7, and 1.0 mg·L^-1^) for root induction. All media were solidified with 7.0 g·L^-1^ agar, supplemented with 30 g·L^ -1^ sucrose, and the pH was adjusted to 5.8. All experiments were repeated three times, treating ten explants at a time. And all experiments were performed at 25 ± 2°C and with a 16 h/day photoperiod with 30 µE m^-2^ of photosynthetically active light.

### Selection of antibiotics concentration for transformation

The differentiating capacity was evaluated using 5 mm × 5 mm leaf pieces. Leaf pieces were cultured in media containing 1.0 mg·L^-1^ BA, 0.3 mg·L^-1^ 2,4-D, 300 mg·L^-1^ Timentin, 300 mg·L^-1^ Carb (Carbenicillin), and various doses of Kan (Kanamycin) (0, 25, 50, 75, 100, 125, 150, and 175 mg·L^-1^) and Hyg (Hygromycin B) (0, 5, 10, 15, and 20 mg·L^-1^), in order to determine the optimal Kan or Hgy concentration for selecting transformed callus. In each Petri dish, 10 leaf discs were grown at each Kan and Hgy concentration, with three duplicates per dish. After 30 days of culture, the growth and development situation and survival rate of explants were recorded.

### Vector construction

The Agrobacterium tumefaciens strain was EHA105 (ANGYUBIO, G6040) and was stored at -80°C. The transformation plasmid was pCAMBIA1301, which contained the CaMv35S-regulated 1,3-glucosidase (GUS) reporter and the neomycin phosphotransferase (NPT‖) gene against kanamycin (Kan). An intron was inserted between the CaMv35S promoter and the GUS gene, which is expressed solely in plant cells.

### Transformation

*Agrobacterium* was incubated in a YEP liquid medium containing antibiotics at 28°C for 36–48 h on a constant temperature shaker at 220 rpm. Then, the bacterial culture was incubated until it appeared clear yellow. Next, 100 mg·L^-1^ AS (Acetosyringone) was added at the appropriate concentration, and the bacterial culture was further incubated for 4 h until OD_600 = _0.6–0.8. Finally, the *Agrobacterium* bacterial culture was centrifuged at 3,000 rpm for 10 min and the supernatant was discarded. A volume of 30–40 mL sterilized distilled water was added to the centrifuge tube. The bacteria were resuspended by gently aspirating with a pipette. Next, the bacterial culture was centrifuged at 7,000 rpm for 5 min at room temperature, this step was repeated once. Finally, the supernatant was poured off, and then about 50 mL of MS liquid medium was added to the centrifuge tube, and it was set aside.

Invitro-raised seedlings grown on MS medium without *PGRs* for 30 d were selected, and the lower middle part of the leaf (Pre-experiments showed a high regeneration rate in the lower and middle part of the leaf) was cut into pieces of 5 mm x 5 mm with scissors. The leaves were inoculated on MS medium containing 1.0 mg·L^-1^ BA and 0.3 mg·L^-1^ 2,4-D and placed in the dark for pre-culture (pre-culture time = 0, 2, 4, and 6 d). Bacterial culture of different OD_600_ (OD_600 _= 0.2, 0.4, 0.6, and 0.8) were added to the Petri dishes followed by pre-cultured leaves for infestation (infection time = 5, 10, 20, and 30 min) with intermittent shaking during the period. Afterward, the bacterial solution was poured off, the excess bacterial solution was blotted off the leaves with sterile filter paper, and the leaves were placed on MS medium containing 1.0 mg·L^-1^ BA, 0.3 mg·L^-1^ 2,4-D, and appropriate concentrations of AS (0, 50, 100, 200 mg·L^-1^) and co-cultured for 3 d at 25°C in the dark. The leaves were transferred to MS medium containing 1.0 mg·L^-1^ BA, 0.3 mg·L^-1^ 2,4-D, 300 mg·L^-1^ Timentin, and 300 mg·L^-1^ Carb for delayed screening (delayed screening time = 0, 2, 4, and 6 d). Afterwards, leaves were transferred to MS medium containing 3.0 mg·L^-1^ tZ, 0.5 mg·L^-1^ IAA, and 10 mg·L^-1^ Hyg for resistance screening. Subculturing was performed every fifteen days and collected after a total of three subculturing. One treatment was repeated five times, for a total of 100 leaves.

### Identification of transgenic-positive callus

After incubation on the selection medium for >45 d, selected resistant calli surviving in the medium were immersed in a GUS staining solution (GUS staining kit, Coolaber SL7160) and held at 37°C for 12 h. The callus samples were decolorized by transferring them into 70% ethanol 2–3 times until the negative control material was white. Successfully stained calli were photographed, and the data was recorded. The genomic DNA was extracted using a Plant DNA Isolation Kit (Fore Gene, DE-06111). GUS-forward (5’-AACCGACGACTCGTCCGTC-3’) and GUS-reverse (5’-GAAGTTCATGCCAGTCCAGCG-3’) primers were used to detect exogenous GUS insertions. PCR was performed using 2×M5 PAGE Taq PCR Mix (Mei5bio, MF047-BD-05) according to the manufacturer’s instructions, and the PCR products were analyzed by agarose gel electrophoresis.

### Statistics and analysis


Rate of callus formation=The number of calli formed/Number of inoculated explants×100%

Regeneration rate=The number of regenerated calli/Number of inoculated explants×100%

Proliferation rate=The number of adventitious shoots/Number of inoculated explants×100%

Rooting rate=The number of shoots with roots/Number of inoculated explants×100%

Genetic transformation frequency=The number of positive calli/Number of inoculated explants×100%

IBM SPSS Statistics 20.0 was used to identify significant differences between treatments (*P*<0.05). All data passed the homogeneity test before Duncan’s multiple range test was applied, and Microsoft Excel software was used to analyze and display the results.

## Results

### Effect of different PGRs on callus induction in ‘Purpurna Saduwa’

**Figure 1:**
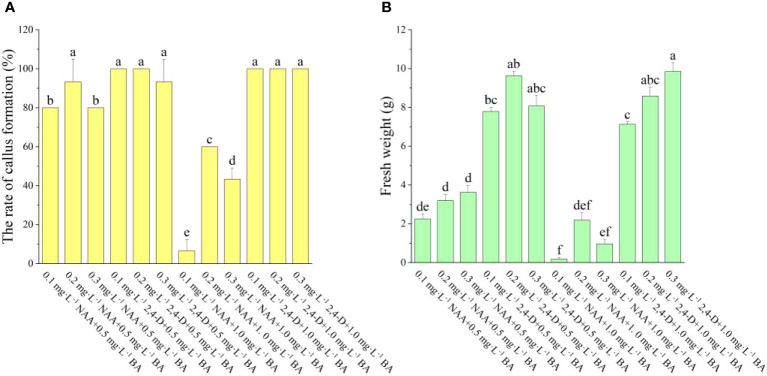
Effect of different PGRs on callus induction in ‘Purpurna Saduwa’ (**A**: the rate of callus formation, **B**: fresh weight). Normal letters in every column indicate significant differences at 0.05 level by Duncan’s multiple range test. The total number was 30 explants. Values represent the mean ± standard error of three replications (n = 10).

**Figure A1:**
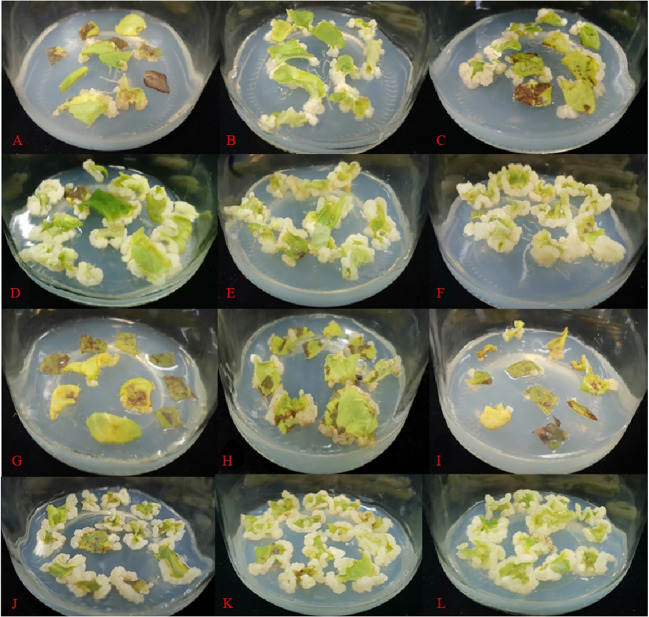
Different *PGRs* combinations of leaf induced callus after 30d (**A**: 0.5 mg·L^-1^ BA + 0.1 mg·L^-1^ NAA, **B**: 0.5 mg·L^-1^ BA + 0.2 mg·L^-1^ NAA, **C**: 0.5 mg·L^-1^ BA + 0.3 mg·L^-1^ NAA, **D**: 0.5 mg·L^-1^ BA + 0.1 mg·L^-1^ 2,4-D, **E**: 0.5 mg·L^-1^ BA + 0.2 mg·L^-1^ 2,4-D, **F**: 0.5 mg·L^-1^ BA + 0.3 mg·L^-1^ 2,4-D, **G**: 1 mg·L^-1^ BA + 0.1 mg·L^-1^ NAA, **H**: 1 mg·L^-1^ BA + 0.2 mg·L^-1^ NAA, **I**: 1 mg·L^-1^ BA + 0.3 mg·L^-1^ NAA, **J**: 1 mg·L^-1^ BA + 0.1 mg·L^-1^ 2,4-D, **K**: 1 mg·L^-1^ BA + 0.2 mg·L^-1^ 2,4-D, **L**: 1 mg·L^-1^ BA + 0.3 mg·L^-1^ 2,4-D).

As shown in **Figure 1**, the various auxins had a greater influence on callus induction. We have observed that the onset time of callus growth induced by different plant hormones varies, with 2,4-D inducing growth earlier than NAA. When the auxin was NAA, the explants began to expand 15 days after inoculation, yet there was no apparent callus, however, when the auxin was 2,4-D, the explants began to swell 10 days after inoculation, and a substantial quantity of calli was evident 15 days after inoculation. When the auxin was NAA, the rate of callus formation declined as BA concentration was increased. When BA concentration was 0.5 mg·L^-1^, there was no significant change in callus formation rate or fresh weight, and when BA was 1.0 mg·L^-1^, there was a significant difference in both callus formation rate and fresh weight among the three treatments. In the medium containing 0.5 mg·L^-1^ BA + 0.2 mg·L^-1^ NAA, the greatest rate of callus development was 93.33%, and the highest fresh weight was 3.62 g·bottle^-1^. When the auxin was 2,4-D, the BA concentration had no significant effect on the rate of callus formation and only a minor effect on the fresh weight. The maximum rate of callus formation (100.00%) and highest fresh weight (9.85 g) was on the medium of 1.0 mg·L^-1^ BA + 0.3 mg·L^-1^ 2,4-D. Moreover, as seen in **Figure 1**, the rate of callus formation and fresh weight induced by 2,4-D were considerably higher than those induced by NAA at the same concentration of BA, indicating that 2,4-D was better at inducing callus than NAA, and thus 2,4-D is more appropriate as an auxin for callus induction in *A. arguta* leaves. In this study, it was concluded that the best combination was 1.0 mg·L^−1^ BA + 0.3 mg·L^−1^ 2,4-D (**Figure A1**).

### Effect of different PGRs on adventitious regeneration of ‘Purpurna Saduwa’

**Figure 2:**
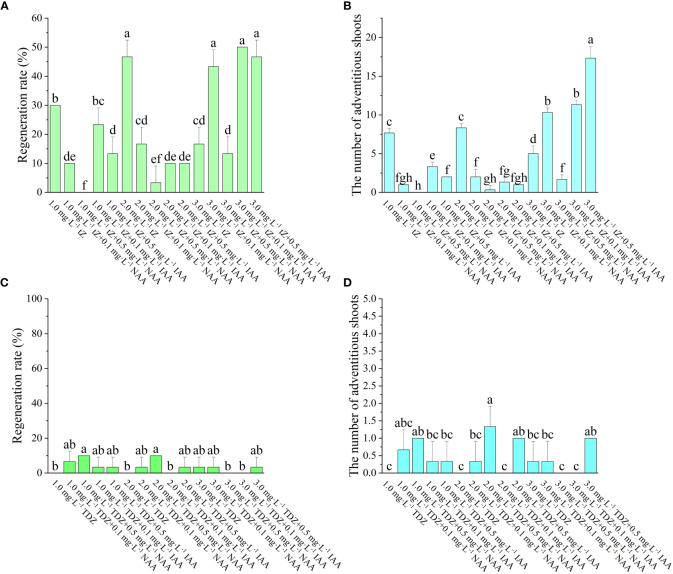
Effect of different *PGRs* on adventitious shoots regeneration of ‘Purpurna Saduwa’(**A, C**: regeneration rate, **B, D**: the number of adventitious shoots). Normal letters in every column indicate significant differences at 0.05 level by Duncan’s multiple range test. The total number was 30 explants. Values represent the mean ± standard error of three replications (n = 10).

According to **Figure 2**, it was found that the induction of adventitious shoots can be achieved even without adding auxin to the medium, as long as tZ is present. As the concentration of tZ increases, both the regeneration rate and the number of adventitious shoots increase. The number of adventitious shoots was greater when tZ was combined with IAA than with NAA. When the auxin was NAA, the regeneration rate increased with increasing tZ concentration and the number of adventitious shoots also showed an increasing trend. When the tZ concentration was the same, the lower concentration of NAA had a higher regeneration rate, with the greatest regeneration rate and the number of adventitious shoots on the medium of 3 mg·L^-1^ tZ + 0.1 mg·L^-1^ NAA at 43.33% and 10.3, respectively. When the auxin was IAA under the same tZ concentration conditions, the regeneration rates of both low and high IAA concentrations were not significantly different and did not change significantly. In the experiment, it was also noted that when IAA was used as the auxin, sprouting occurred earlier than when NAA or no auxin was used. On the medium containing IAA, visible buds appeared after one week of incubation in the light and expanded swiftly into visible seedlings as the incubation period rose, though on the medium containing NAA, visible buds appeared only after two weeks of incubation in the light, and their growth was sluggish. When the tZ concentration was varied over a relatively low range, neither the regeneration rate nor the number of adventitious shoots significantly changed. However, when the tZ concentration was increased to 3 mg L^-1^, both the regeneration rate and the number of adventitious shoots grew substantially. Therefore, the ideal hormone combination for adventitious shoot regeneration was 3 mg·L^-1^ tZ + 0.5 mg·L^-1^ IAA.

The effect of TDZ on the regeneration of adventitious shoots was not significant. The regeneration rate of leaf adventitious shoots was higher for low concentration of TDZ than for high concentration of TDZ. It was observed in the experiment that the differentiation of leaf adventitious shoots was late, and most of them started to appear buds only 8 weeks after inoculation. Meanwhile, the induced adventitious shoots were basically single shoots, and extended incubation time revealed that most of the shoots eventually failed to grow into a robust seedling. Therefore, TDZ was found to be unsuitable as a cytokinin for the regeneration of ‘Purpurna Saduwa’ adventitious shoots in this experiment.

**Figure 3:**
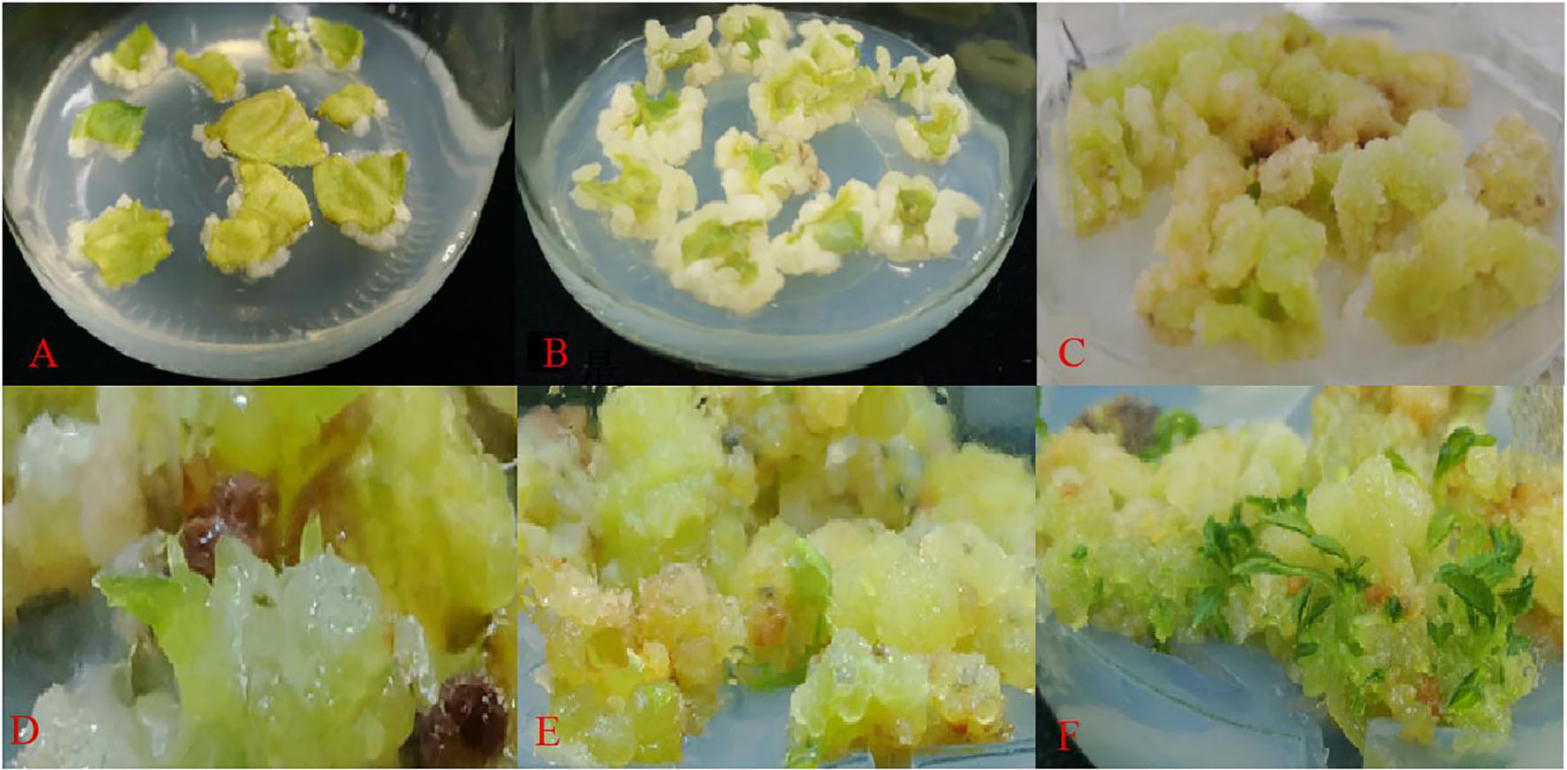
Regeneration of adventitious shoots from ‘Purpurna Saduwa’ leaves (**A**: dark culture for 15 d after inoculation, **B**: dark culture for 30 d after inoculation, **C–E**: light culture for 7 d (37 d after inoculation), **F**: light culture for 25 d (55 d after inoculation)).

The leaves were cultured on a regeneration medium for about 15 d (**Figure 3A**). The leaves began to expand and curl, and there was a small amount of callus at the edge of the leaves. After 30 d of inoculation (**Figure 3B**), a large amount of white callus was observed, the texture of the callus was fluffy. Then, it was transferred to light culture and cultured for 7 d in the light (**Figures 3C–F**), and the texture of the callus became compact from fluffy, and the color changed to bright green. Meanwhile, after 25 d of light culture (**Figure 3F**), adventitious buds could be observed, and with the extension of culture time, the buds gradually developed into a cluster of small plants. And after 45 d of light culture, the adventitious buds were no longer produced and some of the callus began to brown.

### Effect of PGRs on the proliferation of adventitious shoots of ‘Purpurna Saduwa’

**Figure 4:**
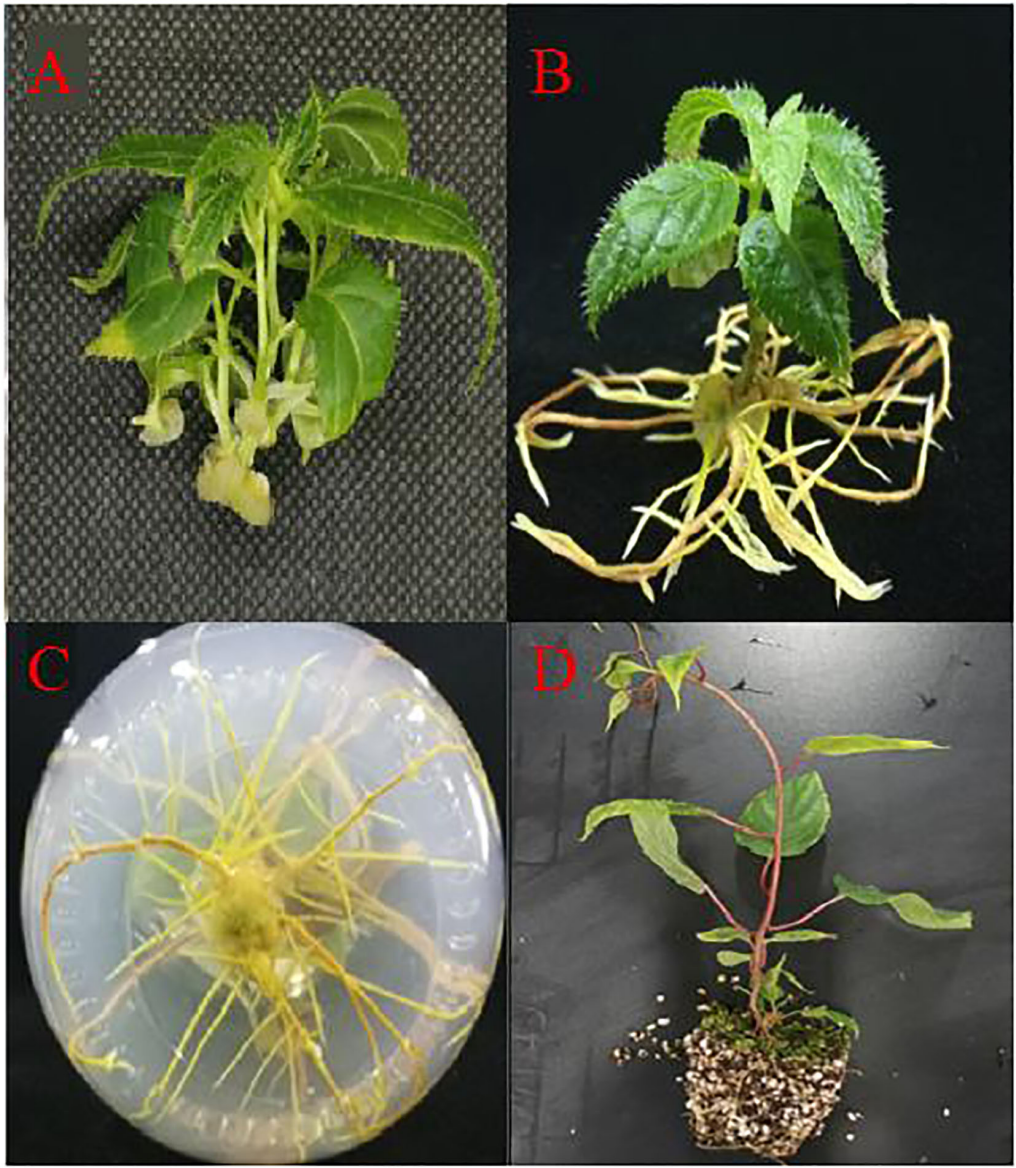
Rapid propagation of kiwifruit ‘Purpurna Saduwa’ (**A**: adventitious bud proliferation; **B, C**: seedling rooting; **D**: transplanting for 30 days).

**Figure 5:**
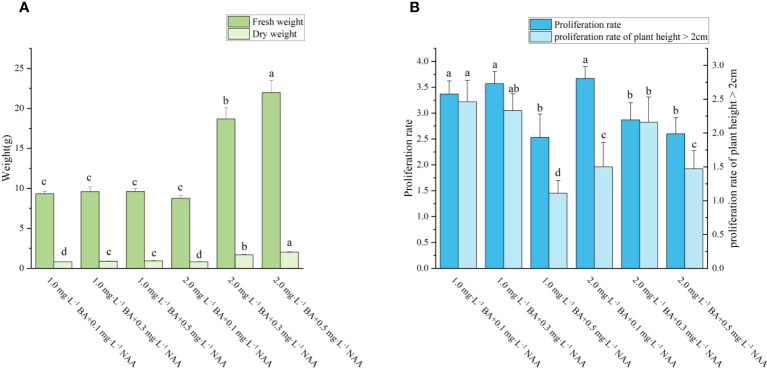
Effects of PGRs on adventitious shoot proliferation of ‘Purpurna Saduwa’(**A**: fresh weight and dry weight, **B**: Proliferation rate and proliferation rate of plant height > 2cm). Normal letters in every column indicate significant differences at 0.05 level by Duncan’s multiple range test. The total number was 30 explants. Values represent the mean ± standard error of three replications (n = 10).

**Figure A2:**
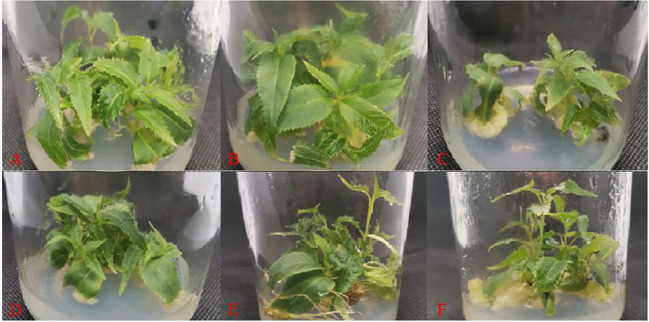
Adventitious shoot proliferation of 'Purpurna Saduwa' (**A**: treatment 1, **B**: treatment 2, **C**: treatment 3, **D**: treatment 4, **E**: treatment 5, **F**: treatment 6).

During the same experiment, it was noticed that adventitious shoots of *A. arguta* were beginning to grow from the axillary buds (**Figure 4A**). According to **Figure 5**, the fresh and dry weights of the latter two treatments were significantly higher than those of the first four treatments. This is likely owing to a substantial amount of callus formation, which encased the original plantlets, inhibiting their growth and resulting in fewer shoots proliferating. Meanwhile, some of the shoots in the latter two treatments displayed malformed leaves with narrow leaves and sharply serrated margins, and they grew excessively long and thin. In treatment 3, the ratio of NAA and BA was near 1:1, which is optimum for callus growth but detrimental for shoot proliferation. The difference between the overall proliferation rates of treatments 1, 2, and 4 was not significant, although treatment 4 resulted in shorter adventitious shoots than the other two treatments. Additionally, the proliferation rate of treatment 4 with a plant height >2 cm was significantly different from treatments 1 and 2. Whereas the difference between treatments 1 and 2 was not significant, and the plantlets proliferated from both treatments grew vigorously, with taller plants and larger leaves (**Figure A2**). Therefore, both treatments 1 and 2 are acceptable for adventitious shoot proliferation.

### Effect of IBA on the rooting of ‘Purpurna Saduwa’

**Figure 6:**
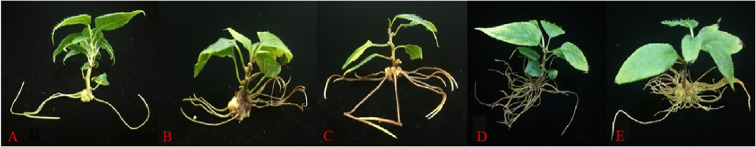
Effects of IBA on rooting of ‘Purpurna Saduwa’ plantlets (**A**: 0 mg·L^-1^ IBA, **B**: 0.3 mg·L^-1^ IBA, **C**: 0.5 mg·L^-1^ IBA, **D**: 0.7 mg·L^-1^ IBA, **E**: 1.0 mg·L^-1^ IBA).

**Figure 7:**
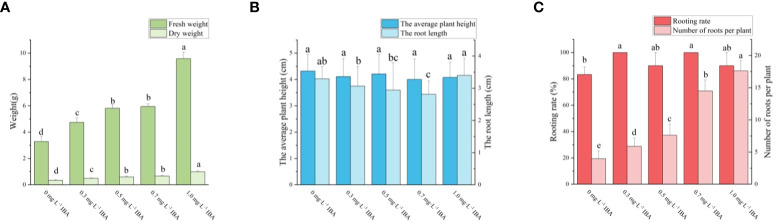
Effects of PGRs on adventitious shoot proliferation of ‘Purpurna Saduwa’(**A**: fresh weight and dry weight, **B**: the average plant height and the root length, **C**: Rooting rate and Number of roots per plant). Normal letters in every column indicate significant differences at 0.05 level by Duncan’s multiple range test. The total number was 30 explants. Values represent the mean ± standard error of three replications (n = 10).

Four days after inoculation, the shoots began to generate callus at the base. A few shoots began to root at seven days. The majority of shoots began to root around 10 days, and after 20 days, no new roots emerged, the roots only became longer (**Figures 4B, C**). It can be seen from **Figure 6**, as the concentration of IBA grew, the amount of callus at the shoots’ bases increased. As can be seen from **Figure 7**, the rooting rate of shoots with IBA addition was higher than that without IBA. The number of roots increases with the increase in IBA concentration, and there are significant differences in the number of roots among treatments with different IBA concentrations. The lowest rooting rate and average root number were 83% and 3.96, respectively, for the medium without IBA addition. When IBA was 1.0 mg·L^-1^, the maximum number of roots was 17.60, but the rooting rate was only 90%. When IBA was 0.3 mg·L^-1^ and 0.7 mg·L^-1^, the rooting rate reached 100%, and the number of roots was 5.90 and 14.50, respectively, and the average number of roots of the latter was significantly higher than that of the former. The fresh weight of shoots also increased with the increase of IBA concentration but in the treatment of IBA at 1.0 mg·L^-1^ (**Figure 6E**), a large number of calli grew at the base of seedlings, resulting in a much higher fresh weight than the other treatments. Considering the rooting rate, root number, and root length, the optimal IBA rooting concentration was 0.7 mg·L^-1^.

### Transplanting

After acclimatization, the plantlets were withdrawn from the vials, the roots were rinsed, and the plantlets were transplanted into a substrate consisting of 7:3 coconut coir: perlite. The pots were properly watered with tap water for the first time. Trays with a 1,000-fold diluted nutrient solution were placed beneath the pots, and the pots were covered with a moisturizing lid to keep them hydrated. A total of 30 seedlings were transplanted and they all survived and flourished (**Figure 4D**).

### Effects of different antibiotics on callus induction of ‘Purpurna Saduwa’ leaves

**Figure 8:**
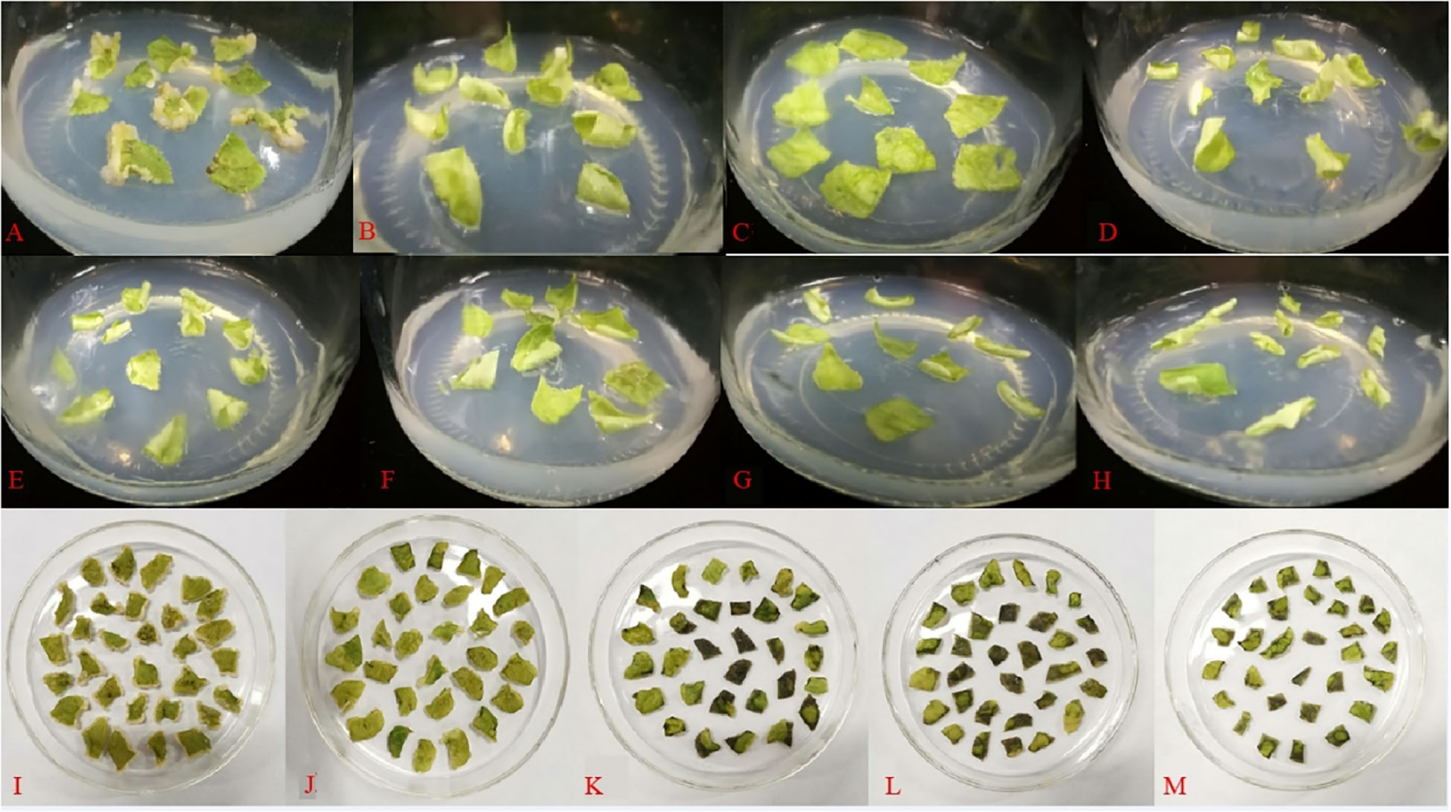
Effect of different antibiotics on the induction of callus in leaves (**A**: 0 mg·L^−1^ Kan, **B**: 25 mg·L^−1^ Kan, **C**: 50 mg·L^−1^ Kan, **D**: 75 mg·L^−1^ Kan, **E**: 100 mg·L^−1^ Kan, **F**: 125 mg·L^−1^ Kan, **G**: 150 mg·L^−1^ Kan, **H**: 175 mg·L^−1^ Kan, **I**: 0 mg·L^−1^ Hyg, **J**: 5 mg·L^−1^ Hyg, **K**: 10 mg·L^−1^ Hyg, **L**: 15 mg·L^−1^ Hyg, **M**: 20 mg·L^−1^ Hyg).

**Figure 8** shows that the leaves were cultivated on antibiotic-free media for 15 days. When the concentration of Kan reached 25 mg·L^-1^, no callus growth was observed on the leaves. As the concentration of Kan was raised, neither callus growth nor browning death was noticed on the leaves. It was thus impossible to determine the deadly concentration of Kan on the leaves. When the antibiotic was substituted with Hyg, the leaves were discovered to be more susceptible to Hyg, with no callus formation at 5 mg·L^-1^ and browning and death of the leaves at 10 mg·L^-1^. Therefore, 10 mg·L^-1^ Hyg was the fatal concentration for leaves and 10 mg·L^-1^ was chosen as the resistance screening threshold concentration.

### Effect of different genetic transformation conditions on the leaf transformation rate of ‘Purpurna Saduwa’

If a single-factor design is used, the best single-factor values are selected one by one, so that the theoretical best combination of conditions selected item by item is not necessarily the true best combination of conditions. If an orthogonal experiment is used, it not only allows for a more scientific experimental design, but also has a balanced dispersion of tests and simple data calculation, as well as reduces workload. As depicted in **Table 1**, the various genetic transformation parameters had a significant impact on the genetic transformation rate, with the greatest rate reaching 21%. Analysis of the range revealed that the magnitude of the effect was in the following order: pre-culture time > AS concentration > infection time > delayed screening time > OD_600_.

**Table 1:** The effect of different factors on the genetic transformation frequency L_16_ (4^5^).

Test number	Pre-culture time/d	OD_600_	Infection time/min	AS concentration (µmol·L^-1^)	Delayed selection time/d	Average genetic transformation frequency
1	0	0.2	5	0	0	1.00%
2	0	0.4	10	50	2	7.00%
3	0	0.6	20	100	4	20.00%
4	0	0.8	30	200	6	12.00%
5	2	0.2	10	100	6	1.00%
6	2	0.4	5	200	4	1.00%
7	2	0.6	30	0	2	3.00%
8	2	0.8	20	50	0	0.00%
9	4	0.2	20	200	2	13.00%
10	4	0.4	30	100	0	21.00%
11	4	0.6	5	50	6	2.00%
12	4	0.8	10	0	4	0.00%
13	6	0.2	30	50	4	2.00%
14	6	0.4	20	0	6	0.00%
15	6	0.6	10	200	0	5.00%
16	6	0.8	5	100	2	8.00%

The optimum level for each treatment was derived by range analysis. As can be seen from **Table A1**, the mean genetic transformation rate for a pre-culture time of 0 d was not significantly different from the mean genetic transformation rate for 4 d, and the mean genetic transformation rate for 2 d was not significantly different from the mean genetic transformation rate for 6 d. The mean genetic transformation rate for the first two treatments was substantially lower than the latter two. Therefore, for the convenience of the experiment, the optimal pre-culture time was 4 d. As can be noted from **Table A2**, AS concentration had a large effect on genetic transformation. When no AS was added to both the culture broth and co-culture medium, the genetic transformation rate was 1.00%, indicating essentially no transgenic callus. The mean genetic transformation rate increased with increasing AS concentration, with the highest mean genetic transformation rate of 12.50% when 100 µmol·L^-1^ AS was added to both the culture broth and co-culture medium. Therefore, the optimum AS concentration was 100 µmol·L^-1^. As shown in **Table A3**, the mean genetic conversion rate tended to increase with increasing infection time. The mean genetic conversion rate was 8.25% when the infection time was 20 min and 9.50% when the infection time was 30 min, the difference between these two mean genetic conversion rates was not significant. Therefore, the best infection time was 20–30 min. As seen in **Table A4**, the average genetic transformation rate reached a maximum of 7.75% at 2 d and 6.75% at 0 d. The average genetic transformation rate at 0 d and 2 d for delayed screening was significantly higher than the average genetic transformation rate at 4 d and 6 d. Therefore, the optimal delayed screening time was between 0 d and 2 d. As can be observed from **Table A5**, the mean genetic transformation rate for OD_600_ of 0.2 and 0.8 was significantly lower than that for 0.4 and 0.6. When OD_600_ was 0.4, the mean genetic conversion rate was 7.25%, when OD_600_ was 0.6, the mean genetic conversion rate was 7.50%, and there was no difference between these two genetic conversion rates. Therefore, the optimal OD_600_ was 0.4 to 0.6.

In summary, the optimal conditions for genetic transformation were: pre-culture for 4 d, OD_600_ of 0.4–0.6, infection for 20–30 min, the addition of 100 µmol·L^-1^ AS to the culture broth and co-culture medium, co-culture for 3 d, and delayed culture screening for 0–2 d.

### GUS-staining of the resistant callus

**Figure 9:**
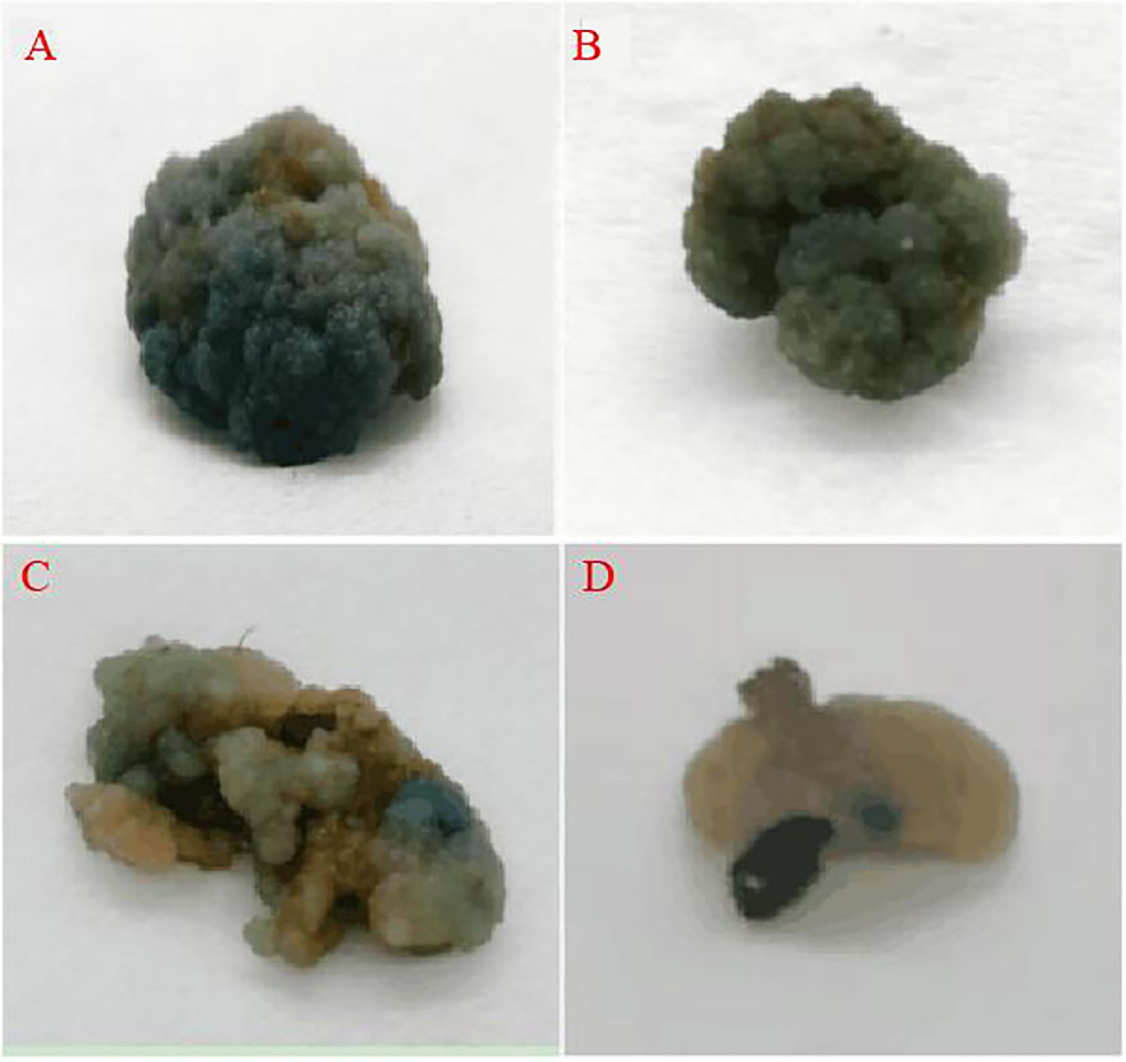
GUS staining of the resistant callus (**A, B**: all blue callus, **C**: partial blue callus, **D**: no blue callus).

**Figure A3:**
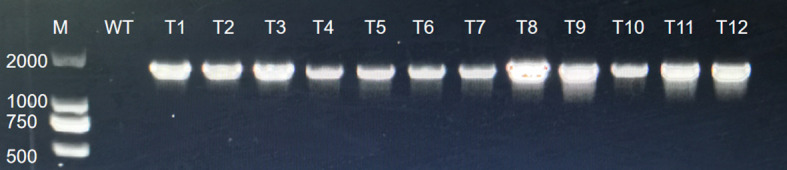
PCR analysis for GUS.

The vector carried the GUS gene, thus, the resistant calli was stained with GUS. Those that were fully stained blue were positive and those that were not stained blue were negative, and the genetic transformation rate was determined based on this staining data and PCR identification. The staining results are presented in **Figure 8**, where it was observed that some of the resistant calli were fully blue (**Figures 9A, B**), others were somewhat blue (**Figure 9C**), and some showed essentially no blue color (**Figure 9D**). The PCR results indicated that the putative transgenic callus contained amplified fragments of the desired size (1800 bp), whereas no amplified fragments were detected for the control callus (**Figure A3**).

## Discussion

### Effect of PGRs on the establishment of regeneration

In plant tissue culture, the type of plant growth regulator and the concentration ratios employed are crucial criteria for experimental success (Wang et al., 2018b). In this experiment, plant growth regulators had a great influence on the formation of calli and plantlet regeneration ability. It was noticed that calli were induced on all 12 mediums, but the induction rate on the BA+2,4-D combination was significantly higher than that of the BA+NAA combination. The fresh weight of the BA+2,4-D combination was 2–3 times higher than the fresh weight of the BA+NAA combination. Browning was present in each treatment of the BA+NAA combination. When BA was 1.0 mg·L^−1^, browning was severe in all three treatments of NAA, indicating that browning was aggravated when BA concentration was too high. Liu et al. found that both high concentrations of BA and low concentrations of NAA aggravated the browning of explants during tissue culture of *A. arguta*, which is similar to the findings of the present study (Liu et al., 2013a). 2,4-D is often used as a callus induction hormone, especially in endosperm callus induction (Dongli et al., 2023). Wang et al. (2018a) and Matkowski and Leslaw (1995) also reached the conclusion that 2,4-D is suitable as a growth hormone for *A. arguta* callus induction.

Trans-Zeatin and BA are the most commonly-used cytokinins for the induction of kiwifruit leaf regeneration, but the appropriate cytokinins in kiwifruit regeneration vary among genotypes. In particular, tZ is frequently used for adventitious shoot induction in kiwifruit callus (Takahashi et al., 2004; Lan et al., 2007). TransZeatin had a desirable effect on the regenerated plants of ‘Purpurna Saduwa’ in this experiment, while TDZ and BA had essentially no effect, which is consistent with the findings of Zhang and Qian (1996) in a study on the leaf regeneration of *A. arguta* plants. Meanwhile, TDZ, as a highly potent cytokinin, has a better effect on the regeneration systems of strawberries and pears. Suezawa et al. (1988) showed that TDZ was more effective than tZ during the differentiation of adventitious shoots from *A. deliciosa*, a finding contrary to the present study, probably due to the difference between the culture and the plant itself. In the present study, it was also discovered that plants could be regenerated only on a medium containing tZ. Therefore, auxin only plays an auxiliary role in the differentiation of explants, and IAA is more effective than NAA. Asakura and Hoshino (2017) also noticed that the use of tZ alone led to shoot regeneration. Therefore, this study shows that tZ is the key hormone in the *A. arguta* regeneration system, and its effect is irreplaceable by TDZ.

In this experiment, it was found that deformed plantlets easily appeared when the BA concentration was 2.0 mg·L^−1^ during the proliferation of adventitious shoots. This observation indicates that ‘Purpurna Saduwa’ is more sensitive to BA and can tolerate lower concentrations of BA. Hu et al. (2019) on Ceratostigma willmottianum Stapf observed that the combination of BA 2.0 mg·L^-1^ and NAA mg·L^-1^ showed the highest proliferation rate of 2.91 for axillary buds. Yu et al., (2014) discovered that the optimal rooting medium for kiwifruit was 1/2MS supplemented with 0.5 mg·L^−1^ IBA, resulting in a rooting rate of 94%. In the ‘Purpurna Saduwa’, it showed a higher propensity for rooting at an IBA concentration of 0.7 mg·L^−1^, which aligns with previous research findings. The best medium for rooting was 1/2MS + 0.5 mg·L^−1^ IBA, with a rooting rate of 94%. The rooting of ‘Purpurna Saduwa’ was easier and the best IBA concentration for rooting was 0.7 mg·L^−1^, which is generally consistent with previous studies (Yu et al., 2014). As a small fruit that is growing in popularity, *A. arguta* has good economic benefits and broad market prospects. With the rise of industrialization of *A. arguta* cultivation, the demand for this high-quality kiwi species has gradually increased, and the establishment of a regeneration system for *A. arguta* is a key solution to this problem. However, the genetic background of *A. arguta* is complex and plant regeneration is difficult. Regeneration systems have been established on only a few kiwi varieties, and the regeneration rates obtained are not high, and stable and efficient regeneration systems are lacking. Through this study, an effective method to improve the quantity and quality of *A. arguta* reproduction was explored. The resulting findings contribute to developing a fast-breeding system for *A. arguta*, provide a theoretical basis for the factory breeding of all-red *A. arguta* plantlets, and offer a way to reduce the breeding cost. *A. arguta*, therefore, may become better used in the food and medicine industries and provide a large amount of high-quality material for research on the genetic transformation system of *A. arguta* as well as its disease resistance, micrografting technology, etc.

### Effect of transformation conditions on conversion rates

Plant transformation plays a key role in plant improvement by introducing beneficial exogenous genes or by silencing the expression of endogenous genes in crop plants (Kumar et al., 2020). Transgenic crops have one or more useful traits, such as herbicide tolerance, insect resistance, abiotic stress tolerance, disease resistance, and nutritional improvement (Rajput et al., 2022). With the development of biotechnology, kiwifruit has also made great progress in genetic transformation, with the translation of some exogenous target genes. Kusaba et al. (1999) transferred the rice homologous gene OSH1, which can alter morphological characteristics, into kiwifruit and obtained transgenic plants exhibiting morphological characteristics such as dwarfism, no apical dominance, small leaves, and leaf margin dehiscence. And the RNA hybridization test showed that the expression of the OSH1 gene in transgenic plants was significantly higher than that of the wild type. Nakamura et al. (1999) transferred a soybeanβ-1,3-endoglucanase gene into kiwifruit and obtained transgenic plants with higher disease resistance than that of the wild type. Kobayashi et al. (2000) successfully transferred the stilbene synthase gene into kiwifruit and obtained transgenic plants. In recent years, more and more exogenous genes have been successfully transferred into kiwifruit. These include ACC (1-Aminocyclopropane-1-carboxylic acid) (Li et al., 2003; Tian et al., 2012), SbtCrylAc (Zhang et al., 2015), and hEGF (Kobayashi et al., 1996).

The Agrobacterium tumefaciens-mediated genetic transformation system is currently the most desirable transformation method for dicotyledonous plants. Its transformation efficiency is often influenced by strain type (Jaiwal et al., 2001; Yadav et al., 2014; Chen et al., 2018), explant pre-culture time (Miguel and Oliveria, 1999; Yao et al., 2013; Verma et al., 2014; Mishra et al., 2016), infection time (Sgamma et al., 2015; Li et al., 2018b; Huang et al., 2017), infection concentration (Coolado et al., 2015; Sun et al., 2017) co-culture time (Tao and Ling, 2006; Wu et al., 2017), and AS concentration and addition method (Singh et al., 2017). Different plants or different parts of the same plant may require unique transformation conditions. In this study, a more systematic transformation condition for *A. arguta* leaf tissue was created.

Kan and Hyg are commonly utilized as screening antibiotics in kiwifruit genetic transformation. Compared to Kan, Hyg inhibits plant development more obviously. For instance, 2–10 mg·L^-1^ Hyg may prevent the development of non-transformed tissue in grapes (Ahmed et al., 2015), pear (Xie et al., 2016), etc. In this investigation, it was determined that 10 mg·L^-1^ of Hyg was the efficient concentration for screening positive callus. This research confirms that pre-culturing for four days can put the explant cells in a state where they are easily able to receive information, which is conducive to the transformation of Agrobacterium and can also reduce the damage caused by Agrobacterium to the explant, thereby increasing the transformation efficiency. This is consistent with the prior findings of kiwifruit genetic transformation studies (Huang et al., 2002; Gao et al., 2007; Liu et al., 2013b). The OD_600_ value was between 0.4 and 0.6, the infection time was between 20 and 30 minutes, and the addition of 100 mol·L^-1^ AS during the Agrobacterium activation stage and co-cultivation improved transformation efficiency, the greatest rate of expression was 21%. In this work, it was also proven that adding AS during the activation stage of Agrobacterium and co-cultivation may considerably enhance transformation efficiency. As infection materials, Lu et al. (2014) utilized the leaves of ‘Huayou’ kiwifruit and discussed the influence of AS addition concentration and addition technique. Consistent with the findings of this investigation, the addition of 75–100 mol·L^-1^ AS to the Agrobacterium activation medium and the co-culture medium simultaneously increased the transformation efficiency. Terakami et al. (2007) observed that the addition of 150 mol·L^-1^ AS to the bacterial solution increased the transformation efficiency of Geranium notably more than other concentrations. In this study, it was noticed that the callus development of infected explants was sluggish. The callus structure was soft, pale in color, and its process of redifferentiation was challenged. Consequently, no transgenic plants were obtained throughout this research. Wang and Wang (2007) saw the same dismal outcomes in their genetic transformation research of kiwifruit, with significant browning of resistant callus and necrosis during the shoot regeneration stage. These researchers suggested that this may be because the explants are too young, and Agrobacterium has a greater virulence effect on young explants (Wang and Wang, 2007). It is possible that one of the reasons for the low regeneration rate is that resistant medium for this experiment was hygromycin. In both maize and bamboo, it was found that the regeneration capacity of the hygromycin-resistant callus was greatly reduced, resulting in plants failing to regenerate (Komari and Kubo, 1999; Ye et al., 2017). Adjusting the medium composition and fostering callus redifferentiation is the next area of study.

Han et al. (2010) obtained the first *A. arguta* transgenic plants with GUS by infecting *A. arguta*‘K2D2’with EHA105 and by reducing the basal salt strength of the medium, based on the results of Wang and Wang (2007), thereby initiating the study of genetic transformation of *A. arguta*. Wang et al. (2012) simultaneously transformed *A. deliciosa, A. chinensis*, *A. eriantha* Benth, and *A. arguta* and demonstrated that the transformation rate and a number of transgenic plants obtained were lowest in *A. arguta* and significantly lower than in the other three species. Herath et al. (2020) Transplanted MYB110 into *A. arguta* and verified the anthocyanin synthesis function of the gene. However, the process did not have a high rate of shoot regeneration, and the genetic transformation efficiency was low. Abdullah et al. (2021) found that regeneration of *A. arguta* was strongly influenced by variety, with significant differences in regeneration rates between varieties. Most of the varieties studied for these studies were green-fleshed *A. arguta* (Li et al., 2015). However, similar studies on all-red-fleshed kiwifruit are scarce (Li et al., 2018a; Lin et al., 2016). These are further evidence of the difficulty of plant regeneration in *A. arguta*. Due to the complicated genetic background of *A. arguta* and the difficulties of plant regeneration, regeneration systems have only been established in a few varieties, and the regeneration rates obtained are low, indicating the lack of a stable and efficient regeneration system. In addition, research on genetic transformation in *A. arguta* has only been conducted for a short length of time. As a result, there is a lack of a stable and comprehensive genetic transformation system. This experiment innovatively established a regeneration system and genetic transformation system for the all-red *A. arguta*. All transplanted plants survived, which is important for the conservation of germplasm resources and practical production applications.

## Data availability statement

The original contributions presented in the study are included in the article/supplementary material. Further inquiries can be directed to the corresponding author.

## Author contributions

WY: Writing - original draft, Visualization, LK: Investigation, Methodology, Data curation, DL: Investigation, Validation, BZ: Methodology, Validation, HT: Software, Methodology, XZ: Investigation, Methodology, YuL: Formal analysis, YTZ: Investigation, QC: Software, YW: Visualization, ML: Investigation, WH: Investigation, YaL: Visualization, XW: Visualization, HRT: Project administration, YZ: Writing - review and editing, Conceptualization, Funding acquisition. All authors contributed to the article and approved the submitted version.

## Appendix A

**Table A1:** Effect of pre-culture time on genetic transformation.

Pre-culture time/d	Average genetic transformation frequency
0	10.00%
2	1.25%
4	9.00%
6	3.75%

**Table A2:** Effect of the AS concentration on the genetic transformation.

AS concentration (µmol·L^-1^)	Average genetic transformation frequency
0	1.00%
50	2.75%
100	12.50%
200	7.75%

**Table A3:** Effect of infection time on genetic transformation.

Infection time/min	Average genetic transformation frequency
5	3.00%
10	3.25%
20	8.25%
30	9.50%

**Table A4:** Effect of delayed selection time on genetic transformation.

Delayed selection time/d	Average genetic transformation frequency
0	6.75%
2	7.75%
4	5.75%
6	3.75%

**Table A5:** Effect of OD_600_ on genetic transformation.

OD_600_	Average genetic transformation frequency
0.2	4.25%
0.4	7.25%
0.6	7.50%
0.8	5.00%

**Table A6:** Reagent information.

Reagent	Catalog number	Company	Country
Trans-tZ	T8110	Solarbio	China
IBA	I538	Phyto Tech	United States
IAA	I885	Phyto Tech	United States
NAA	N600	Phyto Tech	United States
2,4-D	D299	Phyto Tech	United States
BA	B800	Phyto Tech	United States
TDZ	T888	Phyto Tech	United States
Timentin	T8660	Solarbio	China
CarbeniClllin disodium salt	C8251	Solarbio	China
Hygromycin B	H8080	Solarbio	China
Kanamycin	K8020	Solarbio	China
MS Medium	M519	Phyto Tech	United States
GUS stain Kit	SL7160	Coolaber	China
Plant DNA Isolation Kit	DE-06111	Fore Gene	China
EHA105	G6040	ANGYUBIO	China
